# Role of Polygenic Risk Score in Cancer Precision Medicine of Non-European Populations: A Systematic Review

**DOI:** 10.3390/curroncol29080436

**Published:** 2022-08-04

**Authors:** Howard Lopes Ribeiro Junior, Lázaro Antônio Campanha Novaes, José Guilherme Datorre, Daniel Antunes Moreno, Rui Manuel Reis

**Affiliations:** 1 Molecular Oncology Cancer Center, Barretos Cancer Hospital, Barretos 14784-400, SP, Brazil; 2 Center for Research and Drug Development (NPDM), Federal University of Ceara, Fortaleza 60430-275, CE, Brazil; 3 Post-Graduate Program of Pathology, Federal University of Ceara, Fortaleza 60441-750, CE, Brazil; 4 Post-Graduate Program in Translational Medicine, Federal University of Ceara, Fortaleza 14784-400, CE, Brazil; 5 ICVS/3B’s—PT Government Associate Laboratory, 4704553 Braga, Portugal; 6 Life and Health Sciences Research Institute (ICVS), School of Medicine, University of Minho, 4704553 Braga, Portugal

**Keywords:** polygenic risk score, cancer, multiethnic

## Abstract

The development of new screening methods and diagnostic tests for traits, common diseases, and cancer is linked to the advent of precision genomic medicine, in which health care is individually adjusted based on a person’s lifestyle, environmental influences, and genetic variants. Based on genome-wide association study (GWAS) analysis, rapid and continuing progress in the discovery of relevant single nucleotide polymorphisms (SNPs) for traits or complex diseases has increased interest in the potential application of genetic risk models for routine health practice. The polygenic risk score (PRS) estimates an individual’s genetic risk of a trait or disease, calculated by employing a weighted sum of allele counts combined with non-genetic variables. However, 98.38% of PRS records held in public databases relate to the European population. Therefore, PRSs for multiethnic populations are urgently needed. We performed a systematic review to discuss the role of polygenic risk scores in advancing precision medicine for different cancer types in multiethnic non-European populations.

## 1. Introduction

Cancer is amongst the diseases with the highest incidence and mortality, and is considered a worldwide public health problem [1]. In the most recent epidemiological report on the projection of new cancer cases and deaths in the United States, it was estimated that, for the year 2022, a total of approximately 1,918,030 new cancer cases will have been diagnosed, with an estimated 609,360 cases of death for both sexes [2]. The incidence of cancer cases varies significantly between ethnic groups for various reasons, including social issues and inequalities that lead to early detection barriers, prevention and treatment weaknesses [3], the particular genetic characteristics of each population, and differences in individual exposure [4]. Advances in new screening, diagnostic, prevention, and treatment methods for traits or diseases are linked to the advent of precision genomic medicine in which health care is individually adjusted based on a person’s lifestyle, environmental influences, and genetic makeup [4,5].

Population-based genomic screening using genome-wide association studies (GWAS) is at the forefront of a new approach to preventing and establishing precision medicine for traits or diseases such as cancer [5,6]. GWAS studies consider arrays of variants, usually based on analysis of single nucleotide polymorphism (SNP) frequency, which enable identification of associations between genotypes and specific phenotypes and involve testing of differences in allele frequency of genetic variants among individuals who are ancestrally similar and share different traits or diseases [6]. GWAS studies seek to identify and evaluate hundreds or thousands of SNPs that may contribute to risk or operate as protective factors for cancer [7,8,9,10,11,12,13,14].

It is important to note that a major limitation of studies on genetic susceptibility to cancer is the lack of information describing the impact of individuals’ ancestry on risk associations, especially in multiethnic populations [15]. It is noteworthy that some biological and genomic differences may be highlighted as false positives in cross-ethnic analyses, which may have clinical implications for cancer prevention and management in admixture populations [16,17]. Several studies have demonstrated the importance of stratifying admixture populations within case control studies based on genetically well-defined ethnic subgroups (e.g., from the assessment of ancestry-informative markers or ancestry-defining SNPs), such as Africans, Amerindians, Asians, and Europeans [16,17,18]. The importance of a population’s ethnicity with respect to genomic studies can be easily seen when observing the discrepancy in GWAS studies available in public databases [19].

Based on the GWAS catalog [19], 78% of the individuals included in the project were of European origin, 11% were of Asian origin, 2.4% were African, and 1.3% were Hispanic or Latin American [19]. The effects of these ethical discrepancies observed in GWAS studies have been demonstrated and validated in several studies. For example, GWAS data evaluated for individuals with European ancestry should not be extrapolated to all populations with other ethnicities, such as African, Asian, or Latin American, since the risk of traits or cancer may be overestimated [5,20,21]. It is recognized that knowledge of the genomic particularities of each ethnic group may influence the etiology of diseases.

**Figure 1:**
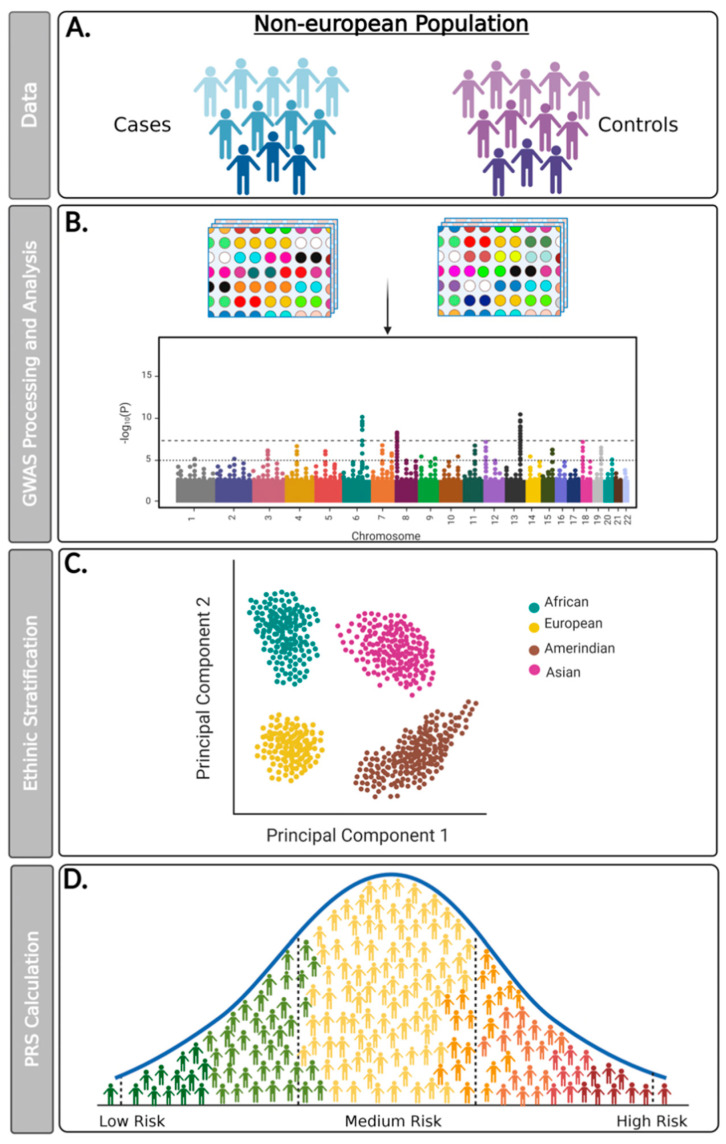
Flowchart detailing proposed steps for conducting GWAS data analysis and PRS calculation for non-European populations. (**A**) Genomic data can be collected from a new study of cohorts developed in multiethnic populations or from genetic information from biobanks or public repositories; (**B**) Genotyping assays must be performed from large-scale microarray platforms and analyzed with reliable bioinformatic tools that use safe quality controls and seek to minimize sampling bias. It is recommended that genotypic data obtained from the GWAS study be paired with information from matched reference populations from repositories such as the 1000 Genomes Project; (**C**) The microarray platform used must encompass genetic variants that allow for the ethnic stratification of the evaluated population (e.g., African, American, Asian, and European); (**D**) The PRS calculation must be performed from an individual’s genetic risk estimate to a trait or disease. The polygenic risk score (PRS) consists of an individual’s genetic risk estimate for a trait or disease, calculated through a weighted sum of allele counts, according to their genotype profile and relevant GWAS data, combined or not with non-genetic variables.

It is known that the individual frequency of allelic variants has minor phenotypic effects on specific traits or diseases in a specific population. However, based on the establishment of mathematical and statistical modeling for GWAS analysis, the simultaneous sum of hundreds or thousands of genetic effects of variants may explain the association between the presence of these variants and the susceptibility and risk stratification of traits or diseases in individuals from well-defined populations [22,23] (Figure 1). Initially proposed by Wray et al. [24], and subsequently widely applied, the polygenic risk score (PRS) consists of an individual’s genetic risk estimate for a trait or disease. The calculation employs a weighted sum of allele counts according to the individual’s genotype profile and relevant GWAS data, combined or not with non-genetic variables (e.g., age, sex, environmental exposure, ancestry or clinical parameters) [23,24,25]. Clinical PRS use is already available for some neoplasms, especially breast cancer [26]. Seeking to normalize and standardize the development of all PRS studies, a recent study by Wand and colleagues [27] emphasized the need to improve reporting standards for polygenic scores in risk prediction studies and to define the minimal information needed to interpret and evaluate PRSs [27].

According to the PolyGenic Score database (PGS Catalog—http://www.pgscatalog.org/) [28] (data available until 21 October 2021), there are a total of 1301 records deposited in the PRS catalog related to 401 traits (cancer or non-cancer) derived from 238 publications. Of the total PRS records deposited in this database, 98.38% (1280/1301) records are related to the European population. Only four (0.39%) PRS registries were established on cancer risk in non-European multiethnic populations [28]. This assessment reflects the emerging need for PRS development in multiethnic populations according to their particular characteristics, highlighting the ethical and clinical differences for each type of disease, especially cancer. Therefore, in the present study, we performed a systematic review to consider the role of polygenic risk scores in advancing precision medicine for different cancer types in multiethnic non-European populations.

## 2. Materials and Methods

### 2.1. Strategy of Research Question Definition

This systematic review was submitted and registered in the OSF database (https://osf.io/gpqxk, accessed on 4 January 2022). The systematic review study was performed according to the Patient or Population, Investigation/Interest, and Context/Outcome (PICO) strategy [29]. The following aspects were included in this systematic review: cancer disease (population), polygenic risk score (investigation/interest), and multiethnic population (context/outcome). According to the three pillars defined for establishing the PICO, it was possible to define the central question of this review as: What is the clinical relevance of polygenic risk scores established for cancer risk stratification in multiethnic non-European populations?

### 2.2. Search the Database

We developed the current systematic review based on the Preferred Reporting Items for *Systematic Reviews* and Meta-Analyses (PRISMA 2020) to establish the minimum evidence necessary to carry out the systematic review about the role of PRSs in cancer precision medicine in multiethnic populations [30]. The authors undertook a systematic search of PubMed/Medline peer-reviewed studies (impact factor greater than or equal to two) published in the last ten years (2011 to 2021). The English-language-based studies were retrieved using the following medical subject headings (MeSH): “*polygenic risk score*” AND “*cancer*” AND “*multiethnic*” OR “*trans-ethnic*” OR “*multiracial*” OR “*ethnic*” OR “*race-ethnicity*” OR “*race*” OR “*mixed population*”. The “*Human*” filter was used to search for articles. Only studies that exclusively used PRSs based on GWAS studies for risk stratification for oncological diseases in multiethnic populations were included in this review. Studies that used data from the UK biobank or European ancestry populations were not considered. Furthermore, only original research studies were included. Review studies, meta-analyses, comments, perspectives, editorials, or other research that did not provide original or unpublished results were excluded. All article records were screened by title and abstract by two independent authors (HLRJ and LACN).

## 3. Results

**Figure 2:**
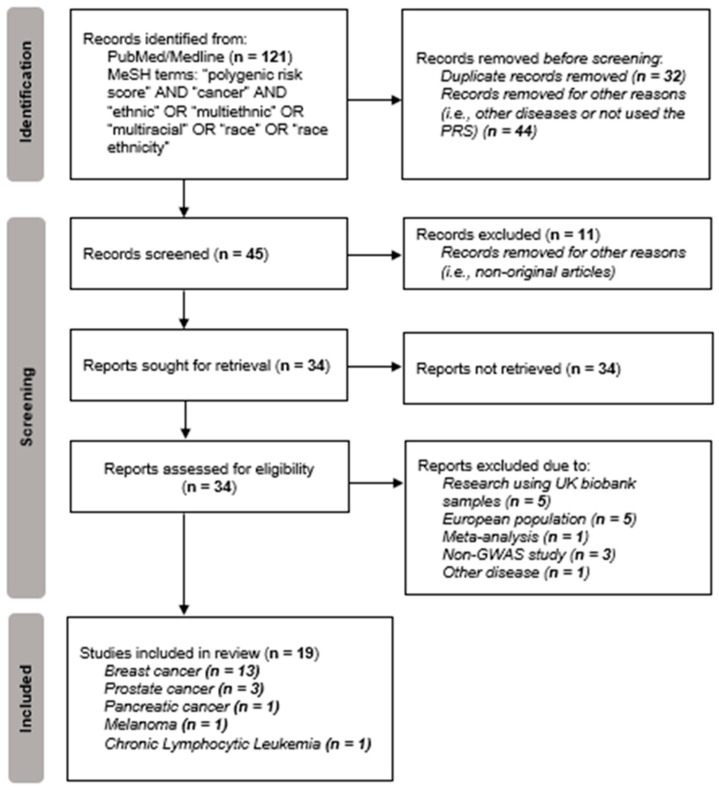
Flowchart of data obtained from the search of PUBMED/Medline records based on the PRISMA methodology [30].

Based on the MeSH terms used, the present systematic review involved the initial retrieval of 121 article records. In the Identification step according to PRISMA [30], 76 articles were excluded because they were duplicates and/or for other reasons, such as studies related to non-cancer diseases or non-original studies (Figure 2). Thus, a total of 45 articles were identified for the screening phase. According to title and/or abstract screening of the 46 articles, eleven non-original articles were excluded. All complete files of articles kept in the screening phase were obtained, to validate their eligibility (*n* = 34). From the remaining total of 34 articles, 15 studies related to research that used samples originating from the UK biobank (*n* = 5) or a European population (*n* = 5), or to a meta-analysis study (*n* = 1), or that were not related to GWAS study (*n* = 3) or were related to other traits or diseases (*n* = 1), were excluded. Finally, 19 articles were included in the systematic review (Figure 2).

Studies that addressed the use of PRS in patients diagnosed with different cancer types (*n* = 19), such as breast cancer (*n* = 13), prostate cancer (*n* = 3), pancreatic cancer (*n* = 1), melanoma (*n* = 1), and chronic lymphocytic leukemia (*n* = 1) were included (Figure 1; Table 1). All 19 articles characterized the association of PRS with disease incidence in multiethnic patients distributed in countries from different continents.

## 4. PRS and the Risk of Breast Cancer

In the USA, breast cancer is the most common form of solid cancer affecting women, with an estimated 43,600 new cases in 2021 [1]. Current GWAS studies have identified hundreds and even thousands of rare variants with moderate to high penetrance, as well as common variants with low penetrance, that contribute to breast cancer risk [31]. However, the need to incorporate common genetic variants into breast cancer risk prediction models has been primarily evaluated in women of European descent.

According to the PGS catalog, breast cancer is the disease with the highest number of PRS records deposited in the database (*n* = 117), with a total of 90 records for the “*breast carcinoma*” index and 17 PRS records developed for specific clinical subtypes of breast cancer (e.g., positive estrogen receptor, negative estrogen receptor, HER2 positive, HER2 negative, Luminal A, Luminal B, and triple-negative breast cancer). Ninety-five percent of the PRS records deposited in the catalog were developed in an exclusively European population. However, only two PRS registries for breast cancer were developed in a non-European population, in this case, in an Asian population. Three PRS registries used a sample of individuals from mixed populations, including Hispanic/Latin American, Asian and African [28]. This systematic review identified 12 studies that evaluated PRS models (e.g., 18-SNP, 53-SNP, 67-SNP, 71-SNP, 75-SNP, 77-SNP, and 143-SNP) for distinct ethnicities (e.g., African, Asian, American, and Hispanic) of breast cancer patients. These data are summarized in Table 1.

**Table 1:** Polygenic risk scores established for breast cancer in multiethnic populations.

Author, Year	Phenotype	Population/ Ethnicity	Subjects (*n*)	SNPs (*n*)	Significant SNPs (*n*)	Main Findings
Evans et al., 2021 [20]	Breast Cancer	Asian Black Jewish Mixed White others Unknown	119 112 120 44 159 274	18-SNP 143-SNP	rs3803662 (*TOX3*) rs2981579 (*FGFR2*)	Not replicated
Allman et al., 2020 [32]	Breast Cancer	African American	Control: 7005 Case: 416	75-SNP	-	Replicated
		Caucasian	Control: 405 Case: 750	77-SNP		
		Hispanic	Control: 3210 Case: 147	71-SNP		
Starlard-Davenport et al., 2018 [33]	Breast Cancer	African-American	Control: 559 Case: 319	75-SNP	-	Replicated
Zhang et al., 2018 [34]	Breast Cancer	American	Control: 7874 Case: 4006	67-SNP	-	Replicated
Shi et al., 2020 [35]	Breast Cancer	Non-Hispanic	Control: 1120 Case: 1152	77-SNP	-	Replicated
Shieh et al., 2020 [36]	Breast Cancer	USA Latin and Latin American	Control: 7622 Cases: 4658	180-SNP	-	Replicated
			Control: 7622 Cases: 4658	71-SNP		
Ho et al., 2020 [37]	Breast Cancer	Asia	Control: 16,483 Case: 15,755	287-SNP	-	Replicated
Hsieh et al., 2017 [38]	Breast Cancer	Asia	Control: 514 Case: 446	6-SNP	rs2981582 (*FGFR2*) rs981782 (*HCN1*) rs889312 (*MAP3K1*) rs3803662 (*TOX3*) rs10822013 (*ZNF365*) rs3784099 (*RAD51B*)	Replicated
Wen et al., 2016 [39]	Breast Cancer	Asia	Control: 11,612 Case: 11,760	44-SNP	rs2046210 (*C6orf97*) rs10822013 (*ZNF365*) rs2363956 (*ANKLE1*)	Replicated
Chan et al., 2018 [40]	Breast Cancer	Asia	Control: 885 Case: 1294 Control: 243 Case: 301 Model 1 Model 2 Model 3	51-SNP 46-SNP 11-SNP 9-SNP	rs16886165 (*MAP3K1*) rs3757318 (*ESR1*) rs11155804 (*ESR1*) rs12662670 (*ESR1*) rs2046210 (*ERS1*) rs10816625 (*CHCHD4P2*) rs704010 (*ZMIZ1*) rs2981579 (*FGFR2*) rs909116 (*LSP1*) rs7297051 (*PTHLH*) rs4784227 (*TOX3*)	Replicated
Coignet et al., 2017 [41]	Breast Cancer	African-American	Control: 744 Case: 621	53-SNP	rs2947411 (*TMEM18)* rs466639 (*RXRG)*	Not replicated
Wang et al., 2018 [42]	Breast Cancer	African	Control: 2029 Case: 1657	34-SNP	-	Not replicated
Wang et al., 2018 [43]	Pancreatic Cancer/Breast Cancer	African	Control: 2029 Case: 1657	23-SNP	rs31490 (*CLPTM1L*) rs40168 (*CLPTM1L*)	Not replicated

*n*, absolute number; SNP, single nucleotide polymorphism. The PRS did (in green) or did not (in red) replicate the risk for cancer in a non-European population compared to a European population.

The discrepancy in the application of PRS studies to breast cancer risk stratification in patients from non-European multiethnic populations has recently been considered. Some authors have demonstrated that PRS models based on 18-SNP, 34-SNP, and 143-SNP models, developed and validated in European populations, cannot be extrapolated to other populations or ethnicities [20,42]. For example, Evans et al. [20] demonstrated that 18-PRSs and a 143-SNP model (previously validated in a White female European population) overestimated the risk of breast cancer in other populations, such as Black, Asian and Jewish populations. Concerning breast cancer patients with African ancestry, Wang et al. [42] investigated whether variants that were risk factors in one population but protective in another (the *flip-flop* effect), originally obtained in extensive studies with European origin women, affected PRS performance in a strictly African population. From the development and evaluation of a 34-SNP-based PRS, the authors identified similar PRS AUC values (0.531) to African patients when compared to a PRS from European ancestry populations (AUC = 0.525) [42]. Thus, these data demonstrate that establishing a PRS based on variants obtained in GWAS studies with European women may have no effect on risk stratification for breast cancer in women of non-European ancestry [42].

Nevertheless, it is important to note that some studies have validated the use of established PRS in European women for breast cancer risk stratification in non-European women [36,37]. Shieh et al. found that a 180-SNP-based PRS was significantly associated with breast cancer risk in Latin women with variable levels of Indigenous American, European, and African ancestry [36]. This finding was also observed when PRS models based on 6-SNP, 46-SNP, 88-SNP, and 287-SNP were evaluated in four different studies with case series of breast cancer patients specifically of Asian ancestry [37,38,39,40].

Asian women with breast cancer with extreme PRS values based on a 287-SNP model had an approximately 2.7-fold increased risk compared to women with intermediate scores [37]. From the application of an 88-SNP-based PRS to 23,567 women with east Asian ancestry, it was found that women within the highest range of PRS values had a significant increase in breast cancer risk (OR = 2.70) when compared to women with PRS values within the 40–60% range [39]. Similarly, based on a 46-SNP model, a PRS demonstrated a significant association in Asian women with respect to the higher (mean = 1.624) and lower (mean = 1.411) quartiles, demonstrating the relevance of PRS in breast cancer risk stratification for this population [40]. Finally, Hsieh et al. [38] developed a PRS based on a specific 6-SNP model and identified a significant distribution of individuals between the highest (OR = 2.26) and lowest (OR < 1.36) quartiles, suggesting that PRS developed with only a few alleles also predict risk of breast cancer in a well-defined population [38]. These results confirm that risk prediction studies established in European populations, with the appropriate development and validation criteria of PRS in multiethnic populations, can, in some restricted situations, estimate the risk of breast cancer in non-European populations.

Several studies have sought to improve established PRSs by adding new clinical or demographic characteristics [32,33,34,35]. Coignet and colleagues [41] reported that the risk assessment established using PRSs based on a 53-SNP model, that were developed initially for European women, was not effective for the risk stratification of breast cancer in African-American patients [41]. Combining five-year and lifetime risks from the conventional BCRAT (*Breast Cancer Risk Assessment Tool*) with a 75-SNP PRS improved the ability to identify risk of breast cancer in a specific African American population in Arkansas in the United States [30]. Interestingly, it was observed that the addition of clinical data of mammographic density and postmenopausal endogenous hormone levels (such as hormonal dosages of testosterone, prolactin, and estrogen) in a PRS developed with a 67-SNP model significantly improved the Gail and Rosner–Colditz risk models for invasive breast cancer in multiethnic US women [31]. There was a significantly decreased association between the PRS based on a 77-SNP model and young-onset breast cancer risk for non-Hispanic women who had ever used hormonal birth control, and a strong association in premenopausal women [32]. A study by Allman et al. [29] supported this hypothesis by demonstrating that adding minimal clinical variables to the PRS (e.g., age and patient’s family history of cancer) could enhance the accuracy and effective prediction of breast cancer risk without diminishing clinical test performance. These results indicate that the use of PRS can improve cancer risk detection using hereditary traits and clinical predictors of breast cancer in non-European populations.

Wang and colleagues [43] evaluated 23-SNPs commonly associated with pancreatic cancer susceptibility in African and European ancestry women with breast cancer. The authors identified that variants rs31490-G and rs401681-T, both from the *CLPTM1L* gene, were related to risk (OR = 1.12) and protective (OR = 0.89) effects, respectively, in African diaspora women with breast cancer, in contrast to what was observed in patients with pancreatic cancer (rs401681-T and rs401681-T OR = 1.20) and women with European ancestry. However, PRS performance based on a 23-SNP model of pancreatic cancer susceptibility variants was not efficient in predicting breast cancer risk in African diaspora women regardless of PRS percentile [43].

Moreover, based on the PRS models evaluated in this systematic review for breast cancer patient risk, we identified the 10 most recurring SNPs, namely rs11249433 (*EMBP1*), rs4973768 (*SLC4A7*), rs10069690 (*TERT*), rs616488 (*PEX14*), rs10941679 (chr5:44706396), rs1432679 (*EBF1*), rs1011970 (*CDKN2B-AS1*), rs704010 (*ZMIZ1*), rs16857609 (*DIRC3*), and rs12493607 (*TGFBR2*) (Supplementary File S1).

## 5. PRS and the Risk of Prostate Cancer

Of the estimated 1,898,160 new cancer cases in the US in 2021, prostate cancer represented the most significant number of estimated cases (*n* = 248,530), significantly surpassing all other neoplastic sites for male cancers [44]. Worldwide, prostate cancer ranks third in newly diagnosed cancer cases [1]. Additionally, prostate cancer constituted a total of 11% (*n* = 34,130) of deaths from cancer in the USA in the same year, being among the leading causes of death, with lung cancer the highest (*n* = 69,410) [44]. The only established risk factors for prostate cancer are age, ethnicity, and family history; however, a significant proportion of prostate tumor cases can also be caused by genetic factors [45].

In a recent review, Bancroft and colleagues [45] provided an update of the current understanding of the impact of polymorphic variants as risk and predisposing genetic factors for the onset of prostate cancer. According to the authors, genetic polymorphisms are present in more than 5% of cases of patients with prostate cancer. However, these variants do not individually increase risk, but, when combined with other variants, are associated with a potentially increased clinically significant risk for prostate cancer susceptibility, as observed in data from European populations [46]. It is important to emphasize the small number of PRS established for the risk of prostate cancer in non-European populations, comprising approximately 6.4% (3/47) of the data presented in the PGS catalog [28].

We present results of PRS development for prostate cancer risk in non-European populations, such as African [47,48] and Latino [46] ethnicities in Table 2. Harlemon et al. [47] showed that a 139-SNP PRS exhibited a much higher prediction risk for prostate cancer in African than in European prostate cancer patients. In addition to heterogeneity between continental ethnicities, intra-ethnic analyzes identified that African subpopulations also demonstrated differences in risk for prostate cancer using the same PRS. For example, individuals from Senegal and Nigeria had a lower and higher predicted risk of prostate cancer, respectively, when compared to other African regions [47]. In an African Ugandan population, Du et al. [48] estimated the effect of known risk alleles using a PRS based on 97-SNPs in prostate cancer patients of this ancestry [42] and observed that the PRS score average was significantly higher for prostate cancer patients when compared to the control group (6.70 versus 6.25). Moreover, men in the top 10% of the PRS distribution had a 2.9-fold elevated risk compared to men included within the 25th––75th PRS percentiles [48]. These results suggest that minimal allele frequency differences in SNPs can contribute to population-level differences in prostate cancer risk in different ethnicities or even within the same ethnicity.

A critical issue for establishing a PRS for a disease is evaluating a multiethnic population, such as the Latino population. Bryc et al. [49] showed that Latinos were extensively admixed from multiple ancestries (e.g., Amerindian, European, and African) and represented < 1% of samples analyzed to date by GWAS cancer projects [46]. Du et al. [46] performed a GWAS study and developed a PRS risk assessment for prostate cancer, based on a 176-SNP model, in 2820 multiethnic Latino patients and 5293 healthy individuals. The patients included in the stratum above 90% and 99% of the PRS had a 3.19-fold and 4.02-fold increased risk, respectively, compared to patients included in the 25th–75th percentiles of PRS. These results demonstrated that PRS could stratify Latino patients based on known-risk SNPs for prostate cancer, indicating an admixture population with distinct genetic variability.

## 6. PRS and the Risk of Pancreatic Cancer

Pancreatic cancer is an aggressive malignant disease that is difficult to detect in the early stages and is related to poor prognosis. By 2021, there were an estimated 60,430 new pancreatic cancer cases in the U.S., with 48,220 deaths forecast for the same year, ranking fourth in the list of deaths by cancer for both sexes [44]. Pancreatic cancer occupies the fourteenth position in the world ranking, with approximately 496,000 new cases and 466,000 deaths in 2020 [1]. Previous studies have described polymorphisms associated with the risk of developing pancreatic cancer [50,51,52,53,54,55]. However, the PRS data on pancreatic cancer in the PGS catalogs were obtained primarily for the European population (*n* = 7) [28].

Nevertheless, a Japanese study evaluated 61 SNPs associated with pancreatic cancer, with five being significantly associated with risk of pancreatic cancer (Table 3). Considering the five SNPs, the PRS calculated values for cases were from 1.17 to 0.42 and for controls were from 1.01 to 0.42, indicating a significant association with pancreatic cancer risk [56]. Future analyses of this PRS should be performed in other populations of patients with pancreatic cancer to validate these results.

## 7. PRS and the Risk of Melanoma

Melanoma is one of the most aggressive types of cancer that affects the world population. For 2020, 325,000 new cases were estimated, with 57,000 deaths [1]. In the U.S., 106,000 new cases were estimated, with 7000 deaths expected for the same year [44]. These numbers highlight the need for faster and earlier diagnosis of risk groups to reduce the number of cases and deaths from the disease. In general, the identification of at-risk groups is based on nongenetic factors; however, adding genetic factors can result in greater efficiency in the identification of at-risk groups [28,58,59,60].

Our meta-analysis identified only one study of the association of PRS with melanoma risk (Table 3) [57]. Cust et al. [57] evaluated the PRS based on a 21-SNP model in two geographically distinct groups of individuals (Australia and the United Kingdom) and observed that those with a high score had a risk three to six times greater than those with a low score in both groups. The odds ratio for PRS was 1.75 for Australians and 1.63 for British people. When adding the PRS to traditional risk factors for melanoma, the AUC increased by 2.3% and 2.8% for Australian and British subjects, respectively [57]. These results suggest that the ancestral component has a greater impact on the frequency of genetic variations than the global regionality of the population, in which groups of the same ancestry have similar PRS values. It is important to note that data on PRS related to melanoma in the PGS catalog are exclusively from the European population, so that ability to appraise the impact of ethnicity is limited [28].

## 8. PRS and the Risk of Chronic Lymphocytic Leukemia

The incidence of chronic lymphocytic leukemia (CLL) was estimated at 21,250 new cases in 2021, corresponding to 25% of new leukemia cases affecting adults over 70 years of age [44]. There is a strong familial relationship with risk of incidence in the population. According to GWAS data, specific inherited polymorphisms are related to CLL development [61,62,63,64,65,66,67,68]. Previous studies have identified SNPs associated with CLL; however, only data on European populations were analyzed in the PGS catalog [28].

Based on 41-SNPs previously described in European individuals [61,62,63,64,65,66,67,68], Kleinstern et al. [21] calculated the PRS in African-Americans (AA) in a cohort of 173 cases and 235 controls, comparing the results with individuals of European ancestry. In this study, two SNPs were significantly associated with risk for CLL, rs7690934 (OR = 1.41) and rs1679013 (OR= 1.56). The average PRS was 7.53 and the OR for CLL was 1.76. The results found in AA were the first to be described for CLL but with a weaker association than observed for EA individuals, with an identified PRS of 8.24 and an OR for CLL of 2.53 [21]. These results suggest that differences based on the ancestry of CLL patients influence the frequency of polymorphisms found in different populations.

## 9. Conclusions

This systematic review evaluated 19 PRS applications for risk establishment for five distinct cancer types, mainly breast cancer, followed by prostate cancer, melanoma, and CLL in non-European populations. We found divergences in risk prediction by PRS established in European versus non-European populations with respect to diagnosis of breast cancer. From independent numbers of SNPs assessed by GWAS studies, significant improvements in risk stratification for prostate cancer, pancreatic cancer, and melanoma were identified, regardless of the ethnic population assessed. However, the PRS reported indicate that CLL risk stratification is a weak predictor of CLL incidence in African-American populations. These data reinforce the importance of the influence of ancestry on genomic composition as a health determinant in non-European populations.

The paucity of GWAS and PRS developmental studies in non-European, and particularly in multiethnic populations, such as Latinos and Brazilians, is remarkable [18]. These populations are underrepresented in genomic studies, leading to important scientific gaps which may negatively impact effective strategies for cancer screening, diagnosis, and prognostication [3,15,69,70,71].

Our group, and others, have made efforts to establish the role of ancestry in the risk stratification and mutational profile of different types of cancers, such as breast cancer [72], lung cancer [73], and colorectal cancer [74] in the Latin American population, specifically from Brazil. The multiethnic profile in the population was demonstrated when it was observed that circa 72% of the patients had a European genetic component, 13% an African component, and 8% and 7% a Native American and Asian component, respectively [72]. Leal and colleagues [73] identified that the somatic mutational frequency of *EGFR* was higher in patients exhibiting a higher Asian genetic component [73]. The similarities identified in the studies presented here, regardless of the type of cancer evaluated, reflect the ethnic admixture of the Brazilian population and the need to assess ancestry to equitably benefit these populations [15].

Despite the findings described, the present study has some limitations, the most important being the paucity of data on the admixture population, limiting PRS adoption and applicability, and exacerbating health disparities. Moreover, there has been a rapid rise in direct-to-consumer assays and for-profit companies (i.e., 23andMe, Color, MyHeritage, and others) that provide PGS and PRS results to customers outside of the traditional patient-provider framework [27], hampering exploration of the data in this review. To overcome this gap, our research group implemented a GWAS study in a large cohort of individuals from a multiethnic Brazilian population to develop a PRS that establishes a possible risk stratification for breast, colorectal, and cervical cancer in Latin America.

Thus, given recent developments in PRS application for cancer precision medicine, there is an urgent need for high-quality research that expands the development of GWAS studies. There is a corresponding need for the stratification of distinct cancer types by PRS in non-European populations to validate the reported PRS and the discovery of new genetic biomarkers, and to improve risk prediction, especially in multiethnic populations.

## Figures and Tables

**Table 2:** Polygenic risk scores established for prostate cancer in multiethnic populations.

Autor, Year	Phenotype	Population/ Ethnicity	Subjects (*n*)	SNPs (*n*)	Significant SNPs (*n*)	Main Finding *
Harlemon et al., 2020 [47]	Prostate Cancer	African	Control: 403 Case: 399	139-SNP	rs183373024 (*PRNCR1*) rs1447295 (*CASC8*)	Not replicated
Du et al., 2018 [48]	Prostate Cancer	African	Control: 485 Case: 571	97-SNP	rs72725854 (none)	Replicated
Du et al., 2020 [46]	Prostate Cancer	Latin	Control: 5293 Case: 2820	176-SNP	-	Replicated

*n*, absolute number; SNP, single nucleotide polymorphism. * The PRS did (in green) or did not (in red) replicate the risk of cancer in a non-European population compared to a European population.

**Table 3:** Polygenic risk scores established for other cancer types in multiethnic populations.

Autor, Year	Phenotype	Population/ Ethnicity	Subjects (*n*)	SNPs (*n*)	Significant SNPs (*n*)	Main Finding
Nakatochi et al., 2018 [56]	Pancreatic Cancer	Asian	Control: 664 Case: 664	61-SNP	rs13303010 (*NOC2L*) rs12615966 (none) rs657152 (*ABO*) rs9564966 (none) rs16986825 (*ZNRF3*)	Replicated
Cust et al., 2018 [57]	Melanoma	Oceania and Europe	Australian: 1035 United Kingdom: 1406	21-SNP	-	Replicated
Kleinstern et al., 2021 [21]	Chronic Lymphocytic Leukemia	African-American	Control: 235 Case: 173	41-SNP	-	Not replicated

*n*, absolute number; SNP, single nucleotide polymorphism. The PRS did (in green) or did not (in red) replicate the risk for cancer in a non-European population compared to the European population.

## Supplementary Materials

The following supporting information can be downloaded at: https://www.mdpi.com/article/10.3390/curroncol29080436/s1, File S1: List of significant SNPs used in the development of PRS to Breast cancer risk stratification in non-European population. Red cells represent the top 10 SNPs included in PRS development.
